# Jay Keystone (1943–2019)

**DOI:** 10.4269/ajtmh.19-1943

**Published:** 2019-10

**Authors:** Isaac I. Bogoch, Sumon Chakrabarti, Abdu Sharkawy

**Affiliations:** 1 Department of Medicine, University of Toronto, Toronto, Ontario, Canada;; 2 Divisions of General Internal Medicine and Infectious Diseases, University Health Network Toronto, Ontario, Canada;; 3 Divisions of Infectious Diseases, Trillium Health Partners, Mississauga, Ontario, Canada

Jay Keystone, beloved father, brother, husband, husband a second time, master clinician, teacher, researcher, and mentor to countless trainees and faculty, has died. Jay will be greatly missed by many, including those he taught and worked with during a career that spanned five decades.

Jay completed his medical school at the University of Toronto (1969), trained in Internal Medicine at the University of Michigan Medical Center, and received a Master’s degree in Clinical Tropical Medicine from the London School of Hygiene and Tropical Medicine (1974). He traveled the world and worked in many settings including West Africa, India, and South America, bringing his growing family in tow.

Jay was one of the early Canadian practitioners of tropical medicine, following in the footsteps of Dr. Michael Lenczner, who established the Tropical Disease Clinic at Toronto General Hospital and Dr. J. D. Maclean, for whom the Centre for Tropical Medicine was named at McGill University in Montreal. He was the go-to clinician for tropical medicine cases; trainees from all over Canada came to work and learn from him. Jay often joked that he taught himself out of a job – later in his career there were fewer referrals to his clinic because most of the Infectious Diseases physicians in the Greater Toronto Area had learned tropical medicine through him. Indeed, Jay will be remembered as a phenomenal and award-winning teacher. He was the recipient of the Ben Kean Medal from the American Society of Tropical Medicine & Hygiene for his excellence in mentoring and teaching (2008). He was awarded the Order of Canada, the highest civilian honor in the country, in 2015, for his outstanding contributions as a pioneer of travel and tropical medicine in Canada.

Jay’s strength was his sense of humor, which he believed was an important tool to ensure that his audience remained engaged, “*an antidote to sleeping sickness”*. He was in his finest form at the weekly Friday morning Tropical Medicine Rounds, which he taught alongside a dedicated band of colleagues known as the “peanut gallery”. It was here where Jay’s humor in teaching shone. When presented with a case of an eschar found on the penis of a febrile traveler recently returned from South Africa, Jay proclaimed a diagnosis “…*Aha! Dick typhus*!”. Or he would lament that his unsteady hand was not ideal for removing *Tunga penetrans* as he felt like he was “*performing a dilatation and curettage on a flea*”. He once discussed an outbreak of traveler’s diarrhea that ravaged the Miami Dolphins football squad with the infamous: “*And that gives new meaning to the term running back!*” Jay would not just toe the line…he would stomp on it while crossing. He once presented what was documented as the most highly attended and highest rated City-Wide Grand Rounds in Toronto, but due to some politically incorrect slides he was given a five-year ban, only to return with another raucous and equally exceptional presentation when the ban was finally lifted. Inevitably, one of Jay’s slides would be a picture of his five children, where he would comment “*I got recreation confused with procreation* ”. He adored his children and would discuss their achievements, and then point out which ones were single and post his phone number if anyone in the audience had a potential suitor. Occasionally people called.

The infectious diseases community of the Greater Toronto Area was deeply saddened when Jay sent a mass email to over eighty people explaining that his “*CT scan from neck to nuts*” had evidence of cancer. But terminal illness could not hold him back, and Jay continued as before with his characteristic commitment and determination in clinic and teaching rounds, even while on chemotherapy. And his mischievous sense of humor never faded; many received an email from Jay with a picture of him standing next to a coffin, with the subject heading: *“Coffin shoppin’!”* Jay remained true to his philosophy of life and wellbeing, and ultimately decided to stop chemotherapy, opting for quality and not quantity of life, but to continue in his roles at the hospital and travel clinic, for therapeutic reasons, for as long as possible. His legacy will live on in the countless trainees, patients, family, and friends whose lives were touched by his extraordinary presence. Jay will be deeply missed.

**Figure:**
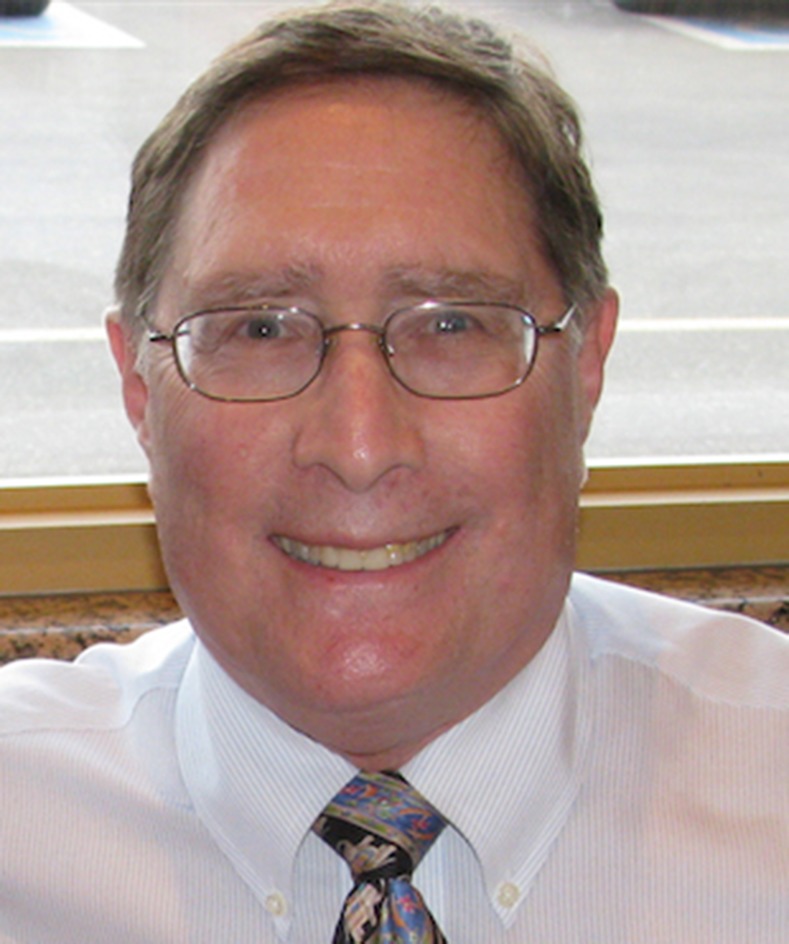
**Jay Keystone**

